# The effect innovation cloning to small business success: entrepreneurial perspective

**DOI:** 10.1186/s13731-022-00245-0

**Published:** 2022-10-05

**Authors:** Harmon Chaniago

**Affiliations:** Department of Business Administration, Bandung State Polytechnic, Bandung, Indonesia

**Keywords:** Innovation cloning, Creativity, Implementation, Owner’s interest, Financial performance, Customer-oriented

## Abstract

It is challenging to run a business during the COVID-19 pandemic, because the owners and the small business leaders must be responsible for success. Leaders need to innovate and look for sources of innovation, including practical inventions, such as innovation cloning. In this study, innovation cloning consisting of creativity cloning and implementation cloning is analyzed for its relationship to business success, in the form of owner’s interest and financial performance perspectives. This study aims to reveal the role of innovation cloning, creativity cloning, and implementation cloning in achieving business success based on the owner’s interest and financial performance. The study uses an explanatory survey. The number of samples is 155 entrepreneurs/small business leaders in Bandung City, Indonesia.  Snowball  sampling is carried out to obtain the data. Data processing uses descriptive statistics and multiple regression with the help of SPSS and Amos 23 software. This study found that creativity cloning and implementation cloning affected the owner’s interest, and implementation cloning affected financial performance. In contrast, creativity cloning does not affect financial performance. Creativity cloning and implementation cloning are correlated with each other. Creativity cloning and implementation cloning are proved to be one unit of cloning innovation. The leader’s expertise in choosing the object to be cloned and adapting it to consumer needs will facilitate company goals. Utilizing innovation, cloning, and modifying it is a practical way for small businesses to succeed. This research needs to be continued to see if there is a continuous pattern in these findings so those findings can be generalized.

## Introduction

Small businesses contribute to the country’s economy and employment (Autio, 2005; Ibarra et al., 2020; Nugroho et al., 2017; Omri et al., 2015; Susanto & Meiryani, 2019). Small business is a form of economic activity that is not too large, and its activities can be in the form of retail, production, trade, and services. In general, small businesses are owned and run by the local communities. The characteristics of a small business are the employees come from immediate families, run independently, using simple technology, and the target market is the local market (Chaniago, 2020; Ingram et al., 2018; Patel et al., 2012).

Several studies have shown that small businesses in developing countries are much higher than medium and large enterprises. Small business grows naturally, and their growth is in line with population growth. Indonesian government divides small businesses into formal and non-formal/micro-groups (UU_No_20, 2008). Non-formal small companies do not have legality and are not registered by the government.

Many small businesses in Indonesia face obstacles in marketing, raw materials, capital, and technology (Ariani & Utomo, 2017; Maksum et al., 2020). Several blocks come from the quality of human resources, which can be overcome by innovation. Innovation becomes an efficient alternative to solve business problems (Pullen et al., 2012). Everyone involved in small businesses, especially owners and leaders, needs to innovate, because innovation will increase business competitiveness. Leaders should provide examples of how to use innovation to solve company problems.

Sources of innovation come from the company’s internal and external environment. The intense business competition encourages the growth of innovation (Chemma, 2021) and forces leaders to try various things, such as studying the business situation. Leaders study, imitate, develop and adapt to consumer needs. Such innovation is also called innovation cloning, and innovation cloning saves research cost development costs and reduces failure risk (TerBraak & Deleersnyder, 2018).

Cloning innovation is a practical way to achieve several goals, such as individual innovation. There are a lot of factors that affect the company’s purpose, and a lot of references stated innovation as the solution. Small businesses will find it hard to innovate unless they clone the innovations of others. Cloning innovation will be more efficient for small businesses, because it is faster and cost less. In its limitations, small business leaders need to be smart in choosing what objects to clone.

There is still little research about individual innovation (Baron & Tang, 2011). Likewise, research on cloning innovations for small business leaders has never been done, and the references are limited. This proves that cloning innovation is still new and has not been studied by researchers. Research on innovation cloning is still rare and only found in the study of TerBraak and Deleersnyder (2018). To the author’s knowledge, no studies have discussed innovation cloning in more detail and applied it to small businesses. Therefore, it is essential to research cloning innovations. This study aims to investigate the cloning activity of leaders’ innovations and analyze the behavior of entrepreneurs in achieving business success. Cloning innovation and business success is largely determined by the leaders or business owners, this is called the entrepreneur perspective.

The results of this study contribute to a specific theoretical framework regarding leadership behavior in innovating, especially cloning innovation in the business world and its benefits for entrepreneurs.

## Literature review

### Cloning innovation

Research on innovation cloning in the business sector is still rare; it can only be found in the study of TerBraak and Deleersnyder (2018). As far as the writer is concerned, there has not been any study discussing innovation cloning that analyzes it into Creativity Cloning (CC) and Implementation Cloning (IC). Each innovation cloning element will be researched and linked to the small businesses’ success.

Researchers often use several innovation terms from the literature, such as radical innovation vs. incremental innovation and original innovation vs. cloning innovation. Innovation radical is defined as a fundamental, comprehensive, and revolutionary change. The characteristics are it tends to make total changes, uses new paradigms, replaces technology, markets, and consumer services. Incremental innovation has changes that occur only in certain parts and are carried out gradually; it is the opposite of innovation radical. Meanwhile, original innovation is defined as the originality of ideas, notions, and plans to be implemented in the company’s organization. Original innovation is hard to find, and the opposite of original innovation is cloning innovation. Innovation cloning is interpreted as imitating various ideas and innovations from outside the company and then modifying them to be applied in their respective companies. Even researchers often do innovation cloning.

Leaders with innovation ability will look for various ideas, adopt them and observe innovations that other people do. This activity is called cloning (TerBraak & Deleersnyder, 2018). In principle, there is no 100% original innovation. Leaders try to perfect and adapt the existing ideas to the conditions and needs of their consumers, so those ideas are suitable for their businesses’ success. In the literature, innovation cloning is concluded as adopting ideas and innovation, adapting to needs, and developing them for implementation in the company (Bhatnagar & Gopalaswamy, 2017; Dubé et al. 2014; TerBraak & Deleersnyder, 2018). This study defines innovation cloning as adopting ideas and notions, imitating and modifying what has been done by other people or successful companies, and implementing them in their own companies. Some activities that are usually cloned are product making, marketing strategies, sales, developing businesses, fulfilling owner wishes, and customer service. This means that innovation cloning is divided into creativity cloning and implementation cloning. TerBraak and Deleersnyder (2018) said that innovation cloning is an attractive strategy with low costs and limited failure risk. Innovative cloning can occur in various company activities, technological cloning and human cloning (Haran et al., 2008), and services to consumers. Cloning activities in the business world can be ideas, notions, or implementation of innovations that other companies have carried out.

In doing innovation cloning, leaders need to be customer-oriented and adjust cloning to their goals. Innovation cloning that often occurs in the business world is related to products and product attributes (TerBraak & Deleersnyder, 2018); products, processes, technologies, organizations and services, markets (Bhatnagar & Gopalaswamy, 2017); service innovation (Berry et al., 2006). Therefore, there are a lot of cloning activities that leaders can do. Looking at the existing explanations and theories, innovation cloning consists of creativity cloning (CC) and implementation cloning (IC). These two variables are used as independent variables.

### Creativity cloning

Creativity is defined as the ability to generate new profitable ideas, combine unique ideas, form new concepts from existing information, identify specific methods to produce new systems and products, and can be applied in any context (Borowski, 2021; Danish et al., 2019; Kassa, 2021). Creativity can make a difference in a company (Márquez & Ortiz, 2021).

Creativity is still a concept; it refers to individual abilities, such as ideas, ideas, and imagination that have not been applied. In discussing creativity, we have to discuss innovation as well. Innovation is ideas or creativities that have been implemented. In its nature, every human being has creativity and innovation inside them. Humans will have the ability to improve a situation if they are willing to use and develop their creativity and innovation.

Innovations come from creativities, such as ideas, notions of the past, or knowledge of people around. People imitate past products and re-engineer them to fit the needs of the present and the next few years. This condition continues in line with time movement and the community’s needs. However, some people do not do it, so they are left behind and are called not innovative, because they do not use their creativity.

According to Jang and Ko (2017), leaders’ creativity includes individual creativity, such as personality, age, and intelligence. Creativity comes from internal and external sources, such as experience passed, the knowledge possessed, personality, and pressure from the environment. Based on the explanation that has been given, creativity is defined as the fruit of human thought and imagination in the form of concepts, ideas, plans, or combinations thereof. Creativity is not yet tangible; it is still a concept. Creativity cloning (CC) imitates and adopts ideas obtained from other people and companies. This research measures CC from idea adoption, development, and new activities.

### Implementation cloning

When an idea or creativity is embodied in action, product, or others, then that creativity is called innovation. Innovation can ensure the company’s survival (Adam & Alarifi, 2021). Innovation is a continuation of creativity. Therefore, innovation is called the implementation of human creativity. Thus, it is essential always to be creative and innovative. Creativity’s value will be known if it is implemented or always used, which means implementing it repeatedly in life.

Meanwhile, implementation cloning (IC) is defined as an activity that imitates successful innovation activities in businesses. IC is measured from products, product attributes, processes, technology, markets, organizations, and services.

### Business success

Leaders have been proven to influence businesses success, as stated in the studies of Amato et. al. (2017); Ibarra et. al. (2020); Cooper (2011); Huang et. al. (2014). Business goals are achieved when the company is successful, which requires clear business success criteria. Currently, there is no agreement on the criteria for business success from researchers (Abu-Rumman et al., 2021; Benzing et al., 2009; Besser & Miller, 2010; Omri et al., 2015). Gorgievski et. al. (2011) proposed using multiple criteria to measure business success. The criteria that can be used are finance, business growth, entrepreneurial goals, consumers, entrepreneur satisfaction, etc.

Benzing et. al. (2009) examined SMEs entrepreneurs in Turkey; their findings concluded that the priority of business success is determined by honesty, friendliness, social skills, and customer service. Those findings illustrate the consumer perspective. From their research in Pakistan, Coy et. al. (2007) concluded that the criteria for small business success are determined by hard work, good customer service, and product quality. This finding illustrates the entrepreneur’s desire or the owner’s perspective. Amato et. al. (2017) classified business success to owner’s financial and satisfaction.

Meanwhile, Walker and Brown (2004) research on SMEs in Australia provided information that financial and non-financial aspects determine business success. According to them, non-financial aspects such as personal satisfaction, pride, independence to be a boss, flexibility of time, and lifestyle are much more valuable. Gorgievski et. al. (2011) has also researched small businesses; the results explained that the criteria for business success are profitability, personal satisfaction, and stakeholder satisfaction. The same thing was found by Simpson et. al. (2012) in their research on the success of the SMEs business; they concluded that factors from the external environment, characteristics of owners and leaders, and organizational characteristics determine business success.

From the existing references and the findings of previous researchers, it can be seen that business success can be seen from three perspectives: (1) Finance, focusing on financial and organizational performance; (2) The interests of the owner and entrepreneur, focusing on the aspirations and desires of the entrepreneur/owner. (3) Consumer needs, focusing on consumer desires, such as product quality, service, honesty, friendliness, price of goods, packaging, guarantees, and discounts (Benzing et al., 2009; Chaniago et al., 2019; Coy et al., 2007).

In short, business success is defined as achieving company goals related to finance, owner’s wishes, and consumer desires within a certain period. To measure a company’s success from three aspects at once (financial, interests of owners, and consumers) takes a lot of time and energy. Because in small businesses, leaders usually double as owners, it is possible to measure business success from the financial–organizational perspective (Fn) and the interests of the entrepreneur (OI) at the same time. Therefore, this study only measures business success from a financial perspective and the owner’s interest. These two variables are used as dependent variables.

### Financial performance

Although business success can be measured from three perspectives, the financial–organizational (Fn) perspective is the most commonly used. This perspective focuses on financial and organizational performance. The financial perspective assumes that business success is only determined by the increase in company assets, such as profit, the number of shares, market share, etc. Khan et. al. (2021) said that the economy and social factors determine business success. In his research, Amato et. al. (2017) proved that business success consists of financial perspective and entrepreneur perspective, such as company age, perceived business success, and company performance (sales, shares, company size). Business success is determined by how far a company’s goal is achieved. However, some literature warns not to measure success from the number of employees, because it can be biased (Walker & Brown, 2004). Small businesses deliberately do not add employees for efficiency, so it is not appropriate to measure small businesses by the number of employees.

The financial–organizational perspective (Fn) sees business success in financial and organizational performance (Amato et al., 2017; Gorgievski et al., 2011; Simpson et al., 2012), such as turnover and profit capital increase, market competition, planning, etc.

### Owner’s interest

Business success can also be seen from the wishes and interests of the owner. Every company owner may have different views and interests, and not all owner interests can be measured financially. Many companies are still maintained in operation by their owners even though they are financially losing money. Various reasons can be identified why the owner maintains it. Measuring sole proprietorship interests is easier, but sole proprietorships are rare. Usually, businesses are run by several people, even though they are still their relatives. However, the goal they want is almost the same. This study seeks to measure the business’s success from the financial side and the owner’s interests. Sometimes company owners want success from other aspects besides finance, for example, social impact, employee welfare, environmental sustainability, good reputation, etc. Measuring success from the perspective of the owner’s interest is called the owner’s interest (OI) perspective. Group of researchers that looks at business success from OI perspective (Coy et al., 2007; Gorgievski et al., 2011; Simpson et al., 2012; Walker & Brown, 2004) set their indicators to owner satisfaction, stakeholder satisfaction, independence, innovative orientation, social impact, self-confidence, flexibility, and lifestyle.

Based on the assumptions and the relationship between innovation cloning of small business leaders (creativity cloning and implementation cloning) and business success, the hypothesis to be tested is formulated as follows:

- H1: Creativity cloning has a positive effect on financial performance.
- H2: Creativity cloning has a positive effect on the owner’s interest.
- H3: Implementation cloning has a positive effect on financial performance.
- H4: Implementation cloning has a positive effect on the owner’s interest.
- H5: Creativity cloning is positively related to implementation cloning.
- H6: Financial performance has a positive effect on the owner’s interest.
- H7: Creativity cloning and implementation cloning simultaneously influence financial performance.
- H8: Creativity cloning and implementation cloning simultaneously affect the owner’s interest.

**Fig. 1:**
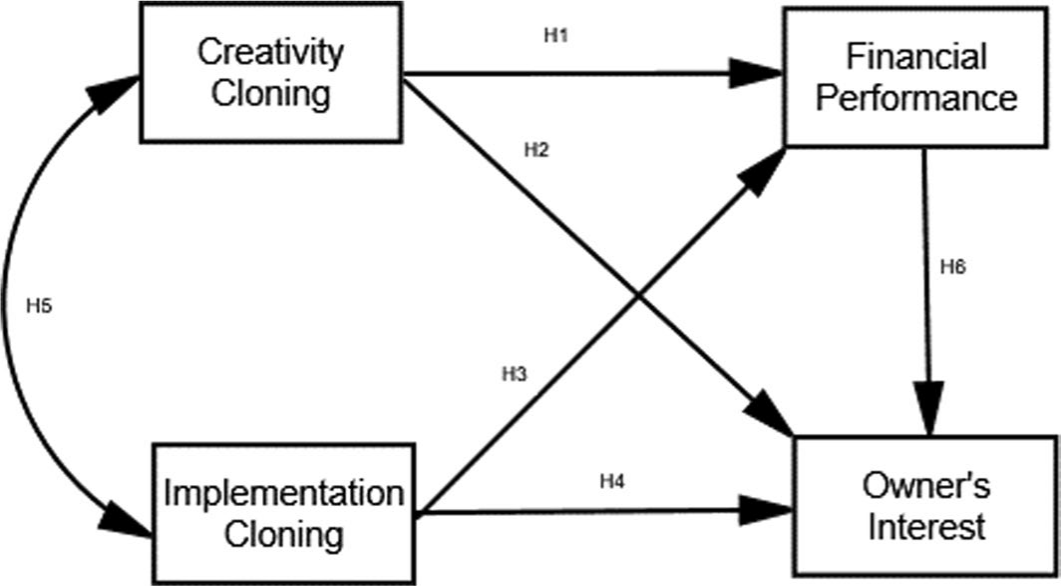
Framework of the study

The relationship between the research concepts is depicted in Fig. 1.

### Research methodology

The research was conducted using an explanatory survey to explain the facts and existing data. The study was carried out in Bandung, Indonesia, in 2021. Bandung is one of the big cities in Indonesia, and it is also a tourist and trading city that is densely populated. In 2019, there were 3793 small businesses in Bandung. Due to the COVID-19 pandemic, 60% will collapse in 2021, leaving only 1516 small businesses (BPS_Kota_Bandung, 2021). This number is used as the research population.

Research respondents are small business owners, because they have a double role as owners and leaders. References from several researchers are used to determine the number of samples. Memon et. al. (2020) said in survey research, “Green’s procedures can be used to determine the number of samples, namely, *n* ≥ 104 + the number of independent variables.” Because there are two independent variables, the minimum number of samples = 104 + 2 = 106 samples. Gay et. al. (2009) suggested that the minimum sample size in descriptive research is 10% of the population; 20% if the population is small. Population 500 is small, and > 500 is a large population (Neuman, 2016). The population of this study is 1,156 small businesses; it is categorized as a large population. Using this assumption, the total sample taken is 10% × 1.516 = 152 and increased to 155 respondents. The total research sample is 155 small businesses, as shown in Table 1.

**Table 1:** Sample distribution on small business

Type of small business	Sum of unit^a^	Sum of the sample (unit)
Retail	411	42
Wholesaling	49	5
Service	147	15
Manufacture	518	53
Other	391	40
Total	1516	155

^a^BPS_Kota_Bandung (2021)

Sampling was done by snowball sampling. This method was carried out to collect respondents with specific criteria. The sample taken is adjusted to the criteria for small businesses according to the Indonesian government constitution, namely, maximum asset value of IDR 500 million (35,000 USD) excluding land and buildings and having a maximum of 20 employees. Other criteria include running for at least 3 years and predominantly operating in Bandung, Indonesia. Only small businesses that meet the criteria are sampled. The list of existing small businesses from the government was contacted via telephone, and some were visited in person.

Some questionnaires were distributed directly to the field and through Google form and social media. Persuasively by phone, small business leaders were reminded to fill out the questionnaires.

The data were processed using descriptive statistics (mean and frequency tests) with the help of SPSS software. This was used to process the demographic data of the respondents. Multiple regression analysis was used to answer the hypothesis with the help of AMOS 23 software. The source of the measurement instrument is presented in Table 2.

**Table 2:** Source of instrumentation

Construct	Source
Innovation cloning	
Creativity cloning (CC):	
Ideas, imagination, Idea adoption	Bhatnagar and Gopalaswamy (2017); Dubé et. al. (2014)
Idea-development, new activity	Björk and Magnusson (2009)
Implementation cloning (IC):	
Product	TerBraak and Deleersnyder (2018); Bhatnagar and Gopalaswamy (2017)
Product attributes	TerBraak and Deleersnyder (2018)
Processes	Bhatnagar and Gopalaswamy (2017); Dubé et. al. (2014)
Technology, market share	Bhatnagar and Gopalaswamy (2017);
Organization and services	Bhatnagar and Gopalaswamy (2017); Berry et. al. (2006)
Business success	
Financial performance-organisation (Fn):	
Capital added, profit, sales, market share, planning	Amato et. al. (2017); Simpson et. al. (2012); Walker and Brown (2004)
Owner’s interest (OI):	
Self-satisfaction, stakeholder satisfaction, social effect, confidence, independence, innovative orientation, flexibilities, and lifestyle	Coy et. al. (2007); Gorgievski et. al. (2011); Walker and Brown (2004); Simpson et. al. (2012)

Source: Compilation of literature, 2021

## Results

Before being used, the data obtained from the respondents were tested for reliability first. The reliability test results showed Cronbach’s Alpha for the CC variable = 0.615; IC = 0.818; Fn = 0.763 and OI = 0.787. All data have Cronbach’s Alpha > 0.6. This means that data obtained are suitable for this study (Gursida & Harmon, 2017; Hair et al., 2010).

The results of descriptive data processing provide information that the majority of respondents are male (59%), are in productive age (30 years), having status as owner and leader (69%). The average education is the graduate degree (70%), and monthly sales are around 1150 USD. The data are shown in Table 3.

**Table 3:** Sample characteristics of SMEs entrepreneur in Indonesia (*N* = 155)

	Frequency	Percent (%)
Entrepreneurial characteristics		
Gender		
Male	92	59
Female	63	41
Average age of entrepreneur (years)	30	58
Level of education		
Graduate degree	108	70
High school	47	30
Entreprise characteristics		
The average age of business (years)	5	67
Type of business		
Retailing	42	27
Wholesaling	5	3
Service	15	10
Manufacture	53	34
Others	40	26
Average of labor/human resources	8	
Occupation		
Manager	48	31
Owner	107	69
Source of capital		
Grant	13	8
Debt	18	12
Own	124	80
Market share		
Local	137	88
National	18	12
Average of sales (USD/month)	1150	
Sales		
Fix	57	37
Decrease	60	39
Increase	38	25

Table 3 shows that 67% of the companies’ age is around 5 years, and the business activities are retail (27%) and manufacture (34%). The workforce uses an average of 8 people. Most small businesses are pretty experienced, the leaders are educated, and 80% use their capital (not loaning from banks). Marketing is still at the local level; as a result, sales are not maximized.

Table 4 shows that the companies leaders scored 3.547 on creativity cloning with a standard deviation of < 1; this is a reasonably necessary criterion. These leaders admit that they sometimes clone ideas, adopt ideas from other parties, imitate how to develop ideas, and make it an activity.

**Table 4:** Mean score for creativity cloning

Indicators	Mean	Standard deviation
Ideas	2.935	1.024
Imagination	4.116	0.868
Idea adoption	4.000	0.845
Idea development	3.335	0.840
New activity	3.348	0.641
Average	3.547	0.843

5 = very-important, 4 = important, 3 = mildly important, 2 = not very-important, 1 = unimportant

Table 5, which consists of 5 indicators, provides information on the average response of small business leaders on implementation cloning (IC). The score is 4.112 with standard deviations < 1. Leaders agree on the importance of implementation cloning, and this score is included in the critical criterion. It indicates that IC is indeed essential for the progress of small businesses. Table 6 shows the average responses of small business leaders on business success from a financial perspective.

**Table 5:** Mean score for implementation cloning

Indicators	Mean	Standard deviation
Product	4.355	0.787
Product of attribute	4.265	0.861
Process	4.226	0.894
Technology	4.084	0.987
Market	3.852	0.952
Organization	3.923	0.957
Services	4.084	0.813
Rata–rata	4.112	0.893

**Table 6:** Mean score business success in financial

Indicators	Mean	Standard deviation
Increase for capital	4.200	0.841
Profit	4.084	0.882
Omzet	3.916	0.868
Market share	3.923	0.957
Planning	4.084	0.813
Average	4.041	0.872

Table 6 measures business success from a financial–organizational perspective (Fn); the total indicators are 5. The average score of respondents’ approval is 4.041, with a deviation of < 1. The explanation shows that respondents agree the achievement of Fn is included in the criteria for success. Furthermore, the processing of descriptive data in Table 7 provides information on respondents’ answers about business success from an entrepreneurial perspective.

**Table 7:** Mean score business success in owner’s interest

Indicators	Mean	Standard deviation
Owner satisfaction	4.355	0.787
Stakeholder satisfaction	4.265	0.861
Social impact	4.123	0.870
Confidence	4.000	0.845
Independence	3.948	0.889
Innovative orientation	4.045	0.784
Flexibylities	4.084	0.897
Life style	4.258	0.844
Average	4.135	0.847

Table 7 shows the mean score of 4.135 and standard deviation < 1; this score is included in the critical or good criteria. It means that entrepreneurs and owners (OI) feel that their goals have been achieved and follow their wishes in a suitable category.

**Fig. 2:**
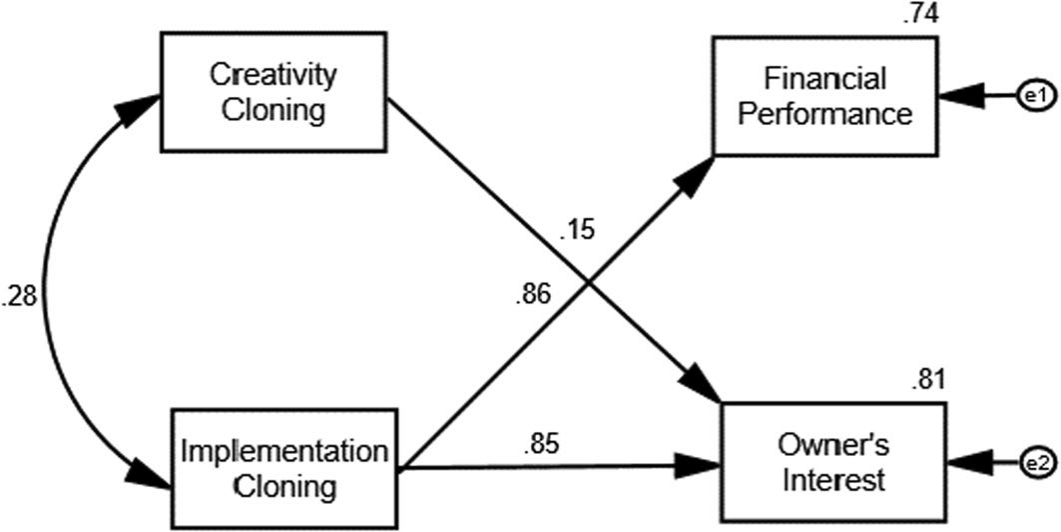
Influence model of innovation cloning and business success

In the next step, data from respondents are processed by multiple regression analysis. With the help of Amos software, the first processing found regression weights (coefficient regression) CC to Fn 0.13 and Fn to OI 0.196. This calculated coefficient is not significant and removed from the calculation. Then, reprocessing is carried out. The results of the second processing are shown in Fig. 2 and Table 8.

**Table 8:** Result of hypotheses test

	*P*	Standardized total effects	Correlations	Squared multiple correlations	Notes
Fn ← CC	0.130	0.842			Rejected H1
OI ← Fn	0.196	− 0.089			Rejected H6
OI ← CC	***	0.151			Accepted H2
Fn ← IC	***	0.861			Accepted H3
OI ← IC	***	0.845			Accepted H4
CC ↔ IC			0.284		Accepted H5
Fn				0.741	Accepted H7
OI				0.810	Accepted H8

***p < 0.01

In Table 8, it can be seen that CC affects Fn 0.842, but not significantly. So the H1 hypothesis is rejected, or it is not proven that creativity cloning influences business success in terms of finance. Likewise, Fn does not affect OI, because the significance level is 0.196 or above 0.05. This information causes hypothesis H6 is rejected, or there is no effect of financial success on the owner’s interest. This study finds that (1) the company’s success in finance does not necessarily satisfy the interests of the company’s owners; (2) creativity cloning does not affect the company’s success from the perspective of financial performance. Table 8 also shows the findings of this study. The results are proof of influential variables, such as:

1. CC affects OI of 15.1%, significant at < 0.05 or accepts the H2 hypothesis. This means that the creativity cloning variable is proven to positively affect the owner’s interest, albeit small.
2. IC affects Fn by 86.1%, significant at < 0.05 or accepts the H3 hypothesis. It means the implementation cloning variable proved to positively affect financial performance, and the effect is categorized as strong criteria.
3. IC affects OI by 84.5%, significant at < 0.05 or accepts the H4 hypothesis. This test proves that implementation cloning also positively affects the owner’s interest, and the effect is included in the strong criteria.
4. CC is correlated with IC by 28.4%, significant at < 0.05, or hypothesis H5 is accepted. The test result proves that creative cloning positively correlates with implementation cloning.
5. CC and IC simultaneously affect Fn by 74.1%, significant at < 0.05, or hypothesis H7 is accepted. This study proves that creativity cloning and implementation cloning positively affect financial performance, and the effect is categorized as strong criteria.
6. CC and IC simultaneously affect OI by 81% significant at < 0.05, or hypothesis H8 are accepted. Simultaneously, creativity cloning and implementation cloning variables also positively impacted the owner’s interest. The influence is included in the strong criteria.

Thus, this study has proven two rejected hypotheses, H1 and H6. The rest of the assumptions, H2, H3, H4, H5, H7, H8, are accepted. The findings of this study show that cloning innovation consisting of CC and IC has a strong effect on OI. Only IC affects the company’s Fn performance, while CC is not proven to affect Fn. It is also proven that CC and IC are correlated. The model testing results using CFA with the help of AMOS software are presented in Table 9.

**Table 9:** Model feasibility test index

No	Criteria	Cut off value	Model result	Explanation
1	Chi-square (*χ*^2^)	Statistics expected small (< table value)	3.938	A small value expected
2	*χ*^2^ Signif. probability	≥ 0.05	0.140	No difference between the data and the model
3	Cr	≤ 2.58	2.408	Normal data distribution
4	CMIN/DF	≤ 2.00	1.969	Good
5	GFI	≥ 0.90	0.988	Good
6	RMSEA	≤ 0.08	0.079	Good
7	AGFI	≥ 0.90	0.938	Good
8	TLI	≥ 0.90	0.988	Good
9	CFI	≥ 0.90	0.996	Good

Source: Hair et. al. (2010)

Table 9 provides information that the feasibility test index of the index value model is > cutoff value. The proposed model is acceptable, and the relationship between the factors is presented in Fig. 2.

## Discussion

This study explains that cloning innovation is seen from two variables: creativity cloning (CC) and implementation cloning (IC). Meanwhile, business success is analyzed from two perspectives: financial–organizational (Fn) and owner’s interest (OI) perspectives. In addition, this study found that CC and IC affect OI either partially or simultaneously. However, Fn is only influenced by IC. The level of influence is included in the strong criteria (Gursida & Harmon, 2017; Hair et al., 2010). This finding provides information that innovation cloning carried out by small entrepreneurs has proven to affect the success of their businesses. This finding also clarifies that innovation cloning is beneficial, especially in carrying out business activities. The benefits of cloning innovation resulting from this study are similar to (Wang et al., 2017) as they used the term absorptive capacity. The term absorbent is identical to cloning innovation. However, this research is more detailed, because it analyzes cloning innovation into two parts: creativity cloning and implementation cloning.

Based on the observations in the field, small business entrepreneurs clone various business ideas and activities from external parties. Clone ideas include product designs, logos, brands, product attributes, processes, technology, markets, and services. Small business leaders can absorb and clone from outsiders, because they are educated and are young. Being young and educated makes it easier to imitate innovations from various places, including the Internet.

They clone and modify the products in demand, such as logos, brands, and packaging, to look almost the same but not identical. Making similar but not the same products are intended to avoid legal aspects. Among small businesses, cloning each other has become a habit. The imitators sell for a relatively low price, and it is a survival and practical way to thrive. Over time, this habit is considered as something natural.

Small entrepreneurs can imitate not all products. They tend to clone products that sell well in the market but are easy to make or do not require special skills and high technology in the making. Cloning products are mainly marketed at the local market. Research demographic data (see Table 3) show that most marketing is at the local level. The average turnover is 1150 USD/month, using eight employees. During the COVID-19 period, small business turnover fell by 39%. The decline in turnover was due to the low purchasing power of consumers and the restrictions on human movement by the government. In developing countries, including Indonesia, innovation cloning is a practical and cost-effective way to produce and compete to get consumers, especially local consumers. Consumers with low purchasing power become their market share. A large market share is one of the factors driving the proliferation of cloning among small businesses.

Another cause is that cloning each other occurs, because small businesses do not have funds for research and development, and the quality of the workforce is still low. Ariani and Utomo (2017); Maksum et. al. (2020) reminded that funds and low quality of human resources are obstacles for small businesses. These constraints encourage leaders to take shortcuts to clone, modify and adapt to their business needs. This is similar to TerBraak and Deleersnyder (2018) research. He concluded that economic motives encourage traditional sellers and retailers to adopt cloning innovations.

The results of the hypothesis analysis show that small business leaders think that CC is quite important. Creativity cloning is related to ideas, imagination, developing ideas, and designing new activities. They can do it and do not entirely depend on cloning from other parties. It is the influence of the leaders’ high education.

The average IC value from the analysis results illustrates that it is included in the essential criteria. It means that entrepreneurs feel that implementing cloning activities about products, product attributes, technology, markets, and services is very important. The company leader admits that it is necessary to follow the example of implementing the implementation of other companies. IC activities determine the success of the business. This research has proven that implementing cloning is essential for small businesses.

Creativity cloning and implementation cloning are innovation cloning activities in the business field. The explanation that has been conveyed shows that both variables are essential. Cloning of innovation occurs as a form of human effort to survive and develop themselves in the economic field. Humans are born to imitate, including in business. The results of data analysis show that CC and IC are correlated with quite strong criteria. This research proves that CC and IC are one unit and are needed to analyze cloning innovation.

The study results also show that CC and IC simultaneously affect business success from the owner’s interest perspective (see Table 8, column 7). The influence is categorized as strong criteria (Gursida & Harmon, 2017; Hair et al., 2010). This implies that CC and IC activities are critical in achieving business success.

The study results on hypothesis testing provide information that only hypotheses H1 and H6 are rejected. The partial effect of CC on Fn is not proven (H1), while the influence of financial performance on OI was not demonstrated. The other six hypotheses are accepted, and their impact is included in the strong criteria; the significant level < 0.05. It proves that CC and IC variables are essential for business success from the owner’s perspective. On the other hand, only the IC variable affects Fn from a financial perspective. The explanation is that IC greatly determines financial performance, such as turnover, profit, capital, and market growth. From the data processing results, this study found that CC and IC are essential variables that can be used to increase business success. Another finding is that IC and CC are proven to be correlated.

For leaders, innovation cloning is a practical way to achieve company goals. Baron and Tang (2011) stated that the owner who doubles as a leader has an excellent opportunity to use various innovations for the company’s success. Leaders easily select, modify and determine which innovations will be used, and he became the center of the company’s progress. In doing innovation cloning, leaders should be oriented to consumer needs (Bhatnagar & Gopalaswamy, 2017; Sundstr€om et al., 2020). Leaders should be selective in doing innovation cloning. It is hoped that the innovations made by the leader can be a factor that encourages the achievement of business success (Omri et al., 2015).

Business success is achieved when the owner’s wishes and the company’s financial targets are accomplished. One of the company’s targets is business sustainability. Business sustainability is related to the circulation of the owner’s funds. The demographics of this research show that most of the funding sources come from internal sources, not using bank funds. The cessation of innovation cloning means the death of small businesses or the loss of owner funds. It is inevitable, and the owner/leader does not like this. In these conditions, cloning innovation becomes the company’s savior.

Another reason is that several large companies also carry out innovation cloning activities, and they even adopt the ideas, works, and imaginations of small business groups. As big businesses have higher funds and better human resources quality, they quickly develop the innovations and then claim them to be original innovations.

This research again reminds all parties that innovation cloning is essential for small businesses to grow and survive. Its contribution is also significant in employment and the country’s economy (Autio, 2005; Ibarra et al., 2020; Nugroho et al., 2017; Omri et al., 2015; Susanto & Meiryani, 2019). It is very unwise to forbid them from cloning in the business world. The solution that policymakers can take in developing countries is to encourage small businesses to improve the quality of their human resources and not to clone identically.

## Conclusions

Cloning innovation consists of creativity cloning and implementation cloning, and both are positively correlated. Business success consists of financial performance and the owner’s interest, and this study found that creativity cloning and implementation cloning positively affected the owner’s interest. Meanwhile, financial performance is only affected by the implementation of cloning. There is no evidence to support that creativity cloning directly affects financial performance. The reason is that creativity is still in the form of a concept and cannot be realized in real terms.

These findings prove that cloning innovation activities can accelerate the achievement of company targets, and this becomes a practical medium for maintaining the sustainability of a business. Cloning innovation activities in small businesses are motivated not only because of economic motives but also for maintaining the continuity of a business. For company leaders, avoid doing similar cloning activities. Try to modify and adapt goods to consumer needs.

## Limitation and future direction

This research was conducted during the COVID-19 period. At this time, many potential SMEs are not operating, resulting in limited data collection. This research should be confirmed again after the COVID-19 pandemic period is over. This research combines all the small business sectors. In the future, it is recommended to do the business sector. Further researchers should also consider increasing the number of samples and expanding the research area in several cities with a large number of SMEs.

## Data Availability

Not applicable.

## Abbreviations

CC: Creativity cloning
IC: Implementation cloning
Fn: Financial
OI: Owner’s interest
IDR: Indonesia Rupiah
USD: United States Dollar
SMEs: Small and medium enterprises
AMOS: Analysis of moment structures
CFA: Confirmatory factor analysis
Cmin: Minimum chi-square statistic
CR: Construct reliability
df: Degrees of freedom
GFI: Goodness-of-fit Index
RMSEA: Root Mean Square Error of Approximation
AGFI: Adjusted goodness-of-fit Index
TLI: Tucker–Lewis Index
CFI: Comparative Fit Index
SPSS: Statistical Package for Social Sciences
